# Biomarker associations with insomnia and secondary sleep outcomes in persons with and without HIV in the POPPY-Sleep substudy: a cohort study

**DOI:** 10.1093/sleep/zsac212

**Published:** 2022-09-14

**Authors:** Nicholas Bakewell, Caroline A Sabin, Riya Negi, Alejandro Garcia-Leon, Alan Winston, Memory Sachikonye, Nicki Doyle, Susan Redline, Patrick W G Mallon, Ken M Kunisaki

**Affiliations:** Institute for Global Health, University College London, London, UK; Institute for Global Health, University College London, London, UK; Centre for Experimental Pathogen Host Research, School of Medicine, University College Dublin, Ireland; Centre for Experimental Pathogen Host Research, School of Medicine, University College Dublin, Ireland; Department of Infectious Disease, Imperial College London, London, UK; UK Community Advisory Board (UK-CAB), London, UK; Department of Infectious Disease, Imperial College London, London, UK; Brigham and Women’s Hospital, Boston, USA; Harvard Medical School, Harvard University, Boston, USA; Beth Israel Deaconess Medical Center, Boston, USA; Centre for Experimental Pathogen Host Research, School of Medicine, University College Dublin, Ireland; Minneapolis Veterans Affairs Health Care System, Minneapolis, USA; University of Minnesota, Minneapolis, USA

**Keywords:** insomnia, sleep problems, HIV, inflammation, biomarkers

## Abstract

**Study Objectives:**

We investigated associations between inflammatory profiles/clusters and sleep measures in people living with HIV and demographically-/lifestyle-similar HIV-negative controls in the Pharmacokinetic and clinical Observations in PeoPle over fiftY (POPPY)-Sleep substudy.

**Methods:**

Primary outcome was insomnia (Insomnia Severity Index [ISI]*>*15). Secondary sleep outcomes included 7-day actigraphy (e.g. mean/standard deviation of sleep duration/efficiency), overnight oximetry (e.g. oxygen desaturation index [ODI]) and patient-reported measures (Patient-Reported Outcomes Measurement Information System (PROMIS) sleep questionnaires). Participants were grouped using Principal Component Analysis of 31 biomarkers across several inflammatory pathways followed by cluster analysis. Between-cluster differences in baseline characteristics and sleep outcomes were assessed using Kruskal–Wallis/logistic regression/Chi-squared/Fisher’s exact tests.

**Results:**

Of the 465 participants included (74% people with HIV, median [interquartile range] age 54 [50–60] years), only 18% had insomnia and secondary sleep outcomes suggested generally good sleep (e.g. ODI 3.1/hr [1.5–6.4]). Three clusters with distinct inflammatory profiles were identified: “gut/immune activation” (*n* = 47), “neurovascular” (*n* = 209), and “reference” (relatively lower inflammation; *n* = 209). The “neurovascular” cluster included higher proportions of people with HIV, obesity (BMI*>*30 kg/m^2^), and previous cardiovascular disease, mental health disorder, and arthritis of knee/hip relative to the other two clusters. No clinically relevant between-cluster differences were observed in proportions with insomnia (17%, 18%, 20%) before (*p* = .76) or after (*p* = .75) adjustment for potential confounders. Few associations were observed among actigraphy, oximetry, and PROMIS measures.

**Conclusions:**

Although associations could exist with other sleep measures or biomarker types not assessed, our findings do not support a strong association between sleep and inflammation in people with HIV.

## Graphical Abstract

**Graphical Abstract:**
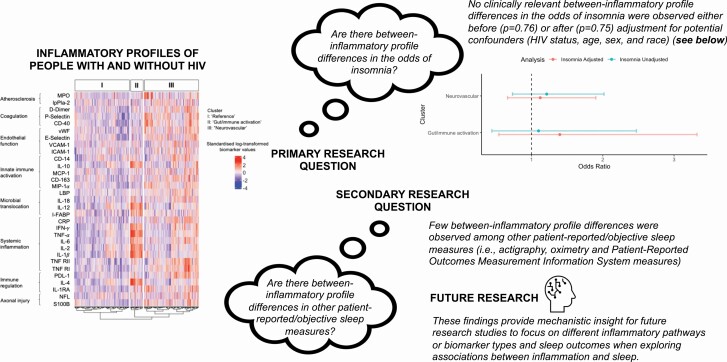

**Statement of Significance**

To the best of our knowledge, this is the first study to explore associations between inflammatory profiles identified in a sample of people living with HIV and demographically/lifestyle similar HIV-negative controls and insomnia as well as secondary objective and patient-reported sleep outcomes. Although we do not report clinically or statistically significant associations between patterns of inflammation and insomnia as well as secondary objective and patient-reported sleep outcomes, these findings provide mechanistic insight that allows us to focus future research studies on different inflammatory pathways or biomarker types (e.g. cerebrospinal fluid), and sleep outcomes (e.g. sleep architecture), when exploring associations between inflammation and sleep.

## Introduction

Poor sleep health is a pervasive public health problem characterized by abnormal sleep duration, sleep disturbances, and/or sleep disorders such as insomnia and sleep apnea. Poor sleep health is often under-recognized and under treated [1–5], despite being treatable and associated with an increased risk of adverse health outcomes, including cardiovascular disease (CVD), diabetes, mental health problems, cognitive impairment, and mortality [4, 6, 7]. The underlying biological mechanisms for how poor sleep contributes to these adverse health outcomes is not yet fully understood, but abnormal immune and proinflammatory responses have been linked to poor sleep health, with biomarkers across several inflammatory pathways (e.g. C-reactive protein (CRP), interleukin (IL)-6, tumor necrosis factor (TNF)-α) proposed to be mediators of the relationship between sleep and adverse health outcomes [7–15].

Sleep problems are commonly reported in people living with human immunodeficiency virus (HIV) and may be associated with socio-demographic and lifestyle factors, side effects of antiretroviral therapy (ART), or direct viral effects [16–18]. Although most people with HIV receiving ART will experience sustained viral suppression, persistent inflammation and immune activation may increase the risk of, and/or exacerbate, sleep problems [16, 17, 19–21]. As the life expectancy of people with HIV increases, age-related physical and mental health comorbidities may further contribute to sleep problems [22, 23].

Insomnia is highly prevalent among people living with HIV and is associated with poorer quality of life, adverse health outcomes and heightened inflammation [16, 19, 24, 25]. People with HIV who report negative impacts of insomnia also tend to experience adherence problems with ART [24, 26, 27]. Therefore, an understanding of insomnia as well as other sleep problems in this group, and their underlying biological mechanisms, is important for the provision of optimal care and guidance in a population with complex health needs.

Using data from the Pharmacokinetic and clinical Observations in PeoPle over fiftY (POPPY)-Sleep sub-study, we previously reported that people with HIV have a higher risk of insomnia (adjusted odds ratio (aOR): 5.3; 95% confidence interval (CI): 2.2, 12.9) than demographically-/lifestyle-similar people without HIV. However, we did not examine potential biological mechanisms for this association [25]. Previous studies have explored associations between inflammatory biomarkers and insomnia and/or other sleep disturbances in people with HIV, generally supporting an association between inflammation and sleep problems [16, 19, 28–31]. However, these studies tended to have small sample sizes, focused on only a few biomarkers, measured insomnia and/or sleep disturbances using general nonvalidated screening questionnaires for sleep disorders, and/or lacked appropriately-selected HIV-negative controls. Here, we determine associations between patterns of inflammation (i.e. inflammatory profiles) and insomnia as well as secondary objective and patient-reported sleep outcomes in people with HIV and lifestyle similar people without HIV.

## Methods

### Study design and participants

The POPPY study is a prospective cohort study that started in 2013 and includes participants from seven clinics in the UK, and one in Ireland. The cohort is comprised of two groups of people with HIV: an older group aged at least 50 years (a priori designed to comprise approximately 50% of the cohort) and a younger group aged 18–49 years (approximately 25% of the cohort) frequency-matched to the older group on sex, ethnicity, sexual orientation and clinic; and one group of demographically-/lifestyle-similar HIV-negative controls aged at least 50 years (approximately 25% of the cohort) frequency-matched to the older group of people with HIV on age, sex, sexual orientation ethnicity and geographical location [32].

Data on demographics, socioeconomic status, anthropometrics, and lifestyle factors were collected at the POPPY baseline visit using self-report forms or recorded by trained clinical research staff, where required. Participants self-reported their clinical history in structured interviews with trained clinical research staff to collect data on comorbidities and/or clinical conditions that are present, medications that have been received and any healthcare resources that have been used over the past year; hospital notes were also reviewed to validate the presence of comorbidities [32, 33]. Details on the comorbidities and clinical conditions assessed and their classification in the POPPY study have been previously published [33].

A subset of 483 POPPY participants was recruited into the POPPY-Sleep substudy with a similar proportion of participants to the overall cohort in HIV and age groupings. Participant selection for the substudy was independent of previously reported sleep symptoms but was based on additional inclusion criteria that a participant be able/willing to wear a fingertip oximetry device and wrist actigraph for a week and, based on the investigator’s judgment, able to adhere to study procedures.

Participants attended one study visit between March 2017 and July 2018, where they completed questionnaires on sleep quality, symptoms for sleep disorders, sleep medical history and medication use for sleep problems. Assessment of anthropometric measurements and cognitive function was also conducted during the visit. The study visit was followed by in-home procedures including a daily sleep diary, actigraphy and oximetry measurements, and another visit to return completed sleep diaries and devices. In addition, fasting plasma samples were collected from participants at or near the time of POPPY-Sleep substudy enrollment and stored in ultra-low temperature freezers for biomarker assessments.

All participants provided written informed consent and the study was approved by the UK Health Research Authority & Research Ethics Committee (number 16/LO/2175) and local ethics committee and/or institutional review boards.

### Biomarker procedures

The laboratory analysis was performed at the Centre for Experimental Pathogen Host Research (CEPHR), University College Dublin (UCD) on frozen ethylenediaminetetraacetic acid (EDTA)-derived plasma samples. The 31 biomarkers analyzed cover 8 inflammatory pathways based on their biological function: atherosclerosis [Myeloperoxidase (MPO), Lipoprotein-associated phospholipase A2 (Lp-PLA2)]; coagulation [D-Dimer, P-Selectin, Cluster of Differentiation (CD)-40]; endothelial function [von Willebrand factor (vWF), E-Selectin, Vascular Cell Adhesion Molecule 1 (VCAM-1), Intercellular Adhesion Molecule 1 (ICAM-1)]; innate immune activation [CD-14, IL-10, Monocyte Chemoattractant Protein (MCP)-1, CD-163, Macrophage Inflammatory Protein (MIP)-1, CD-163, Macrophage Inflammatory Protein (MIP)-1α]; microbial translocation [Lipopolysaccharide-binding Protein (LBP), IL-18, IL-12, Intestinal fatty-acid binding protein (I-FABP)]; systemic inflammation [CRP, Interferon (IFN)- γ TNF-α, IL-6, IL-2, IL-1 β, TNF receptors I and II (TNF RI and TNF RII)]; immune regulation [Programmed death-ligand (PDL)-1, IL-4, IL-1receptor antagonist (IL-1RA)]; and axonal injury [Neurofilament light chain (NFL), S100B].

The biomarkers were analyzed using two platforms that are immunoassays based on Enzyme Linked Immunosorbent Assay (ELISA). One platform was Meso Scale Discovery (MSD; Rockland, MD, USA) that uses electrochemiluminescence for analyte detection. MSD’s immunoassay multiplexing kit (Cat no. K15067L-2) was used to measure 10 biomarkers (TNF-α, IFN-γ, IL-1β, IL-1RA, IL-2, IL-4, IL-6, IL-10, IL-12, IL-18) and antibody sets (Cat no. F214E-3, F214C-3, F217X-3) were used to develop assays for MPO, PDL-1, and NFL, respectively. I-FABP was also analyzed on the MSD platform with an ELISA development kit from R&D Systems (Minneapolis, MN, USA; Cat no. DY3078). The other platform used to measure the remaining 17 biomarkers was Luminex—MAGPIX (Luminex, R&D Systems, Minneapolis, MN, USA) that uses a magnetic bead-based multiplex assay that detects analytes by chemiluminescence. Two customized multiplexing immunoassay kits (Cat no. LXSAHM-14 and LXSAHM-03) from R&D Systems were used to measure 14 biomarkers (MCP-1, CD-40, D-Dimer, Lp-PLA2, E-Selectin, TNF RI, TNF RII, VCAM-1, MIP-1α, CD-163, ICAM-1, S100B, P-Selectin, vWF) and 3 biomarkers (CRP, LBP, and CD-14), respectively.

Single-use plasma aliquots were utilized for each biomarker to avoid the effect of freeze–thaw cycles. Samples were run in duplicate along with control and standard curves on each plate, and the coefficient of variation (CV) was used as a quality control criterion. Any sample with an intra-plate CV above 10% and inter-plate CV above 15% was checked against control and standard curves, and repeated. Any participant that had a biomarker measurement with unreliable CVs above the previously specified thresholds even after 2 to 3 repeats was flagged as having an unreliable biomarker measurement and removed from analyses.

### Sleep outcomes

#### Primary outcome: insomnia.

The primary outcome of this study was insomnia determined from the results of the Insomnia Severity Index (ISI), a validated self-administered screening questionnaire [34]. The ISI consists of seven Likert-type items regarding symptoms of insomnia occurring in the previous 2 weeks each measured on a scale from 0 to 4. Responses for all seven Likert-type items were summed resulting in a total ISI score ranging from 0 to 28. For this study, participants with an ISI score *>*15 were classified as having insomnia in line with Bastien et al. [34].

#### Secondary sleep outcomes: patient-reported and objective sleep measures.

Secondary patient-reported sleep outcomes determined *a priori* were T-scores from the Patient-Reported Outcomes Measurement Information System (PROMIS) sleep disturbance (-SD) and sleep-related impairment (-SRI) 8-item short-form questionnaires [35]. Raw scores from the PROMIS questionnaires were transformed into T-scores, where a population mean score is 50 and standard deviation (SD) is 10. Higher T-scores indicate greater sleep disturbance or sleep-related impairment [35].

Actigraphy measurements were obtained from a wrist-worn actigraph device (ActiGraph wGT3X-BT, ActiGraph Corporation) to estimate physical activity and sleep/wake periods. Participants wore the device continuously from the study enrollment visit until the time of return (at least 7 days later), except for times contraindicated to avoid damaging the device (e.g. bathing, swimming). Oximetry measurements were obtained from an in-home overnight pulse oximetry using a battery-powered, wrist-worn overnight fingertip pulse oximetry device (WristOx 3150; Nonin Medical, Plymouth, MN) with high-resolution sampling (1 sample per second) throughout a single night. Raw actigraphy and oximetry data were downloaded at the study site and electronically transferred to the Sleep Reading Center (Brigham and Women’s Hospital, Boston, MA) for central quality control review. Actigraphy data were scored centrally, blinded to HIV status, after review of the raw actigraphy data and data obtained from sleep diaries to annotate time in bed, main sleep period, and napping. The mean and the SD of actigraphy measures were calculated across the 7-day observation period for each participant. Oximetry data recordings were annotated to identify periods of likely sleep period and artifact; retesting requests were made when <3 hours of artifact-free data were found. After annotations to the oximetry data, records were processed to generate oxygen saturation metrics. Data collection procedures for actigraphy and oximetry data for the POPPY-Sleep sub-study have been previously described in greater detail [25, 36].

From the actigraphy data, secondary objective sleep outcomes were defined *a priori* as the mean and SD (i.e. measures of night-to-night variability) of: wake after (main) sleep onset (WASO), length of awakenings, number of awakenings, sleep duration, sleep maintenance efficiency, sleep onset latency, and movement index. Secondary objective sleep outcomes identified *a priori* from oximetry measurements were the Oxygen Desaturation Index (ODI) (4% desaturation) and percentage of time with oxygen saturation (SpO2) below 90% (i.e. time hypoxic, or sleep-related hypoxia/hypoxemia).

### Statistical analysis

We adopted methodology from a previous study that utilized Principal Component Analysis (PCA) followed by unsupervised agglomerative hierarchical cluster analysis (AHCA) to identify inflammatory profiles in high-dimensional biomarker data collected from people with HIV and HIV-negative controls in Ireland [37]. In our study, PCA was carried out on the 31 biomarker variables collected from participants to reduce the dimensions of the data, only including data from participants with reliable biomarker measurements not flagged during the biomarker procedures. Prior to conducting PCA, biomarker variables were log-transformed for approximate normality because PCA was carried out on the correlation matrix, which requires data to satisfy univariable and bivariate normality assumptions of the Pearson’s correlation coefficient. PCA was conducted on the correlation matrix because the variability differed across biomarkers and the correlation matrix treats all biomarkers on an equal footing [38]. The number of PCs to retain was determined by examining the Scree plot using the Elbow method. PC scores were calculated for participants using the linear combination of PC loadings for each of the PCs retained.

Following PCA, unsupervised AHCA was carried out using the Ward’s minimum variance method and the squared Euclidean distance as the distance measure to group participants into clusters with distinct biomarker patterns based on their PC scores. Conducting PCA prior to AHCA was done not only to reduce the dimensions of the data but also as a denoising method to improve the reliability of the number of clusters identified. The average silhouette width (ASW) method was used to determine the optimal number of clusters [39]. A heatmap displaying the clusters (i.e. inflammatory profiles) identified was created using the log-transformed standardized biomarker measurements to visually explore distinct patterns in biomarker measurement values across clusters.

In this present analysis, data were used from both the POPPY baseline and POPPY-Sleep substudy visits. Baseline characteristics, sleep medication use for insomnia and associations of cluster membership with insomnia (ISI ≥ 15), actigraphy/oximetry measures, and sleep-related impairment and disturbance (PROMIS-SRI/-SD T-scores) were assessed using Kruskal–Wallis tests for continuous outcomes or logistic regression, Chi-squared and Fisher’s exact tests for categorical outcomes, as appropriate. Likelihood ratio tests (LRTs) were used to test for an overall association between the clusters identified and insomnia for the logistic regression analysis of insomnia before and after adjustment for potential confounders. Among people with HIV, between-cluster differences in CD4+ T-cell count and percentages with viral suppression (HIV-RNA *<* 50 copies/mL) and on ART were assessed using Kruskal–Wallis, Chi-squared, and Fisher’s exact tests, as appropriate. Confounder variables included in the adjusted logistic regression for the insomnia analysis were identified *a priori* as HIV status, age, sex, and race. Profile likelihood-based CIs are reported instead of Wald CIs to adjust for the potential presence of sparse data bias in the insomnia analysis due to a relatively small number of participants with insomnia per variable. Missing data were handled using listwise deletion, excluding participants who were missing data on any variable included in an analysis. No adjustment for multiple testing was made in this study. We instead emphasize the clinical relevance, interpretation of the size of associations and associated CIs, and consistency of results across related sleep outcomes.

Additional details on the assessment of model fit, sensitivity analyses on potential sparse data bias, adjustment for additional observed potential confounders, missing data, use of the raw ISI score as a continuous outcome, exclusion of HIV-negative controls or people with HIV who were experiencing viraemia (HIV-RNA > 50 copies/mL), and analyses of each biomarker separately are provided in the Supplementary Material.

All analyses were performed using R version 4.1.0, with two-sided *p*-values < .05 considered to be statistically significant [40].

## Results

### Participant characteristics

Of the 483 participants enrolled in the POPPY-Sleep substudy, 475 participants had plasma samples tested, of whom 465 participants had reliable biomarker measurements and were included in the analyses (343 (74%) people with HIV, median (interquartile range [IQR]) age 54 [50–60] years), most were male (80%), men who have sex with men (MSM) (71%) and white (88%). Few (8.4%) participants reported sleep medication use for insomnia at the POPPY-Sleep substudy visit. The median [IQR] time between the POPPY baseline visit and POPPY-Sleep substudy visit was 9 [2–17] months; and median [IQR] time between the POPPY-Sleep substudy visit and plasma sampling collection date was 8 [7–11] days. Among those with HIV, most (98%) were on ART, 314 (92%) had HIV-RNA*<*50 copies/mL and median [IQR] CD4+ T-cell count was 610 [470–785] cells/mm^3^. The 10 participants excluded from analyses because of unreliable biomarker values did not differ in any meaningful way from those included in analyses.

### Principal component analysis and agglomerative hierarchical cluster analysis

**Figure 1.**
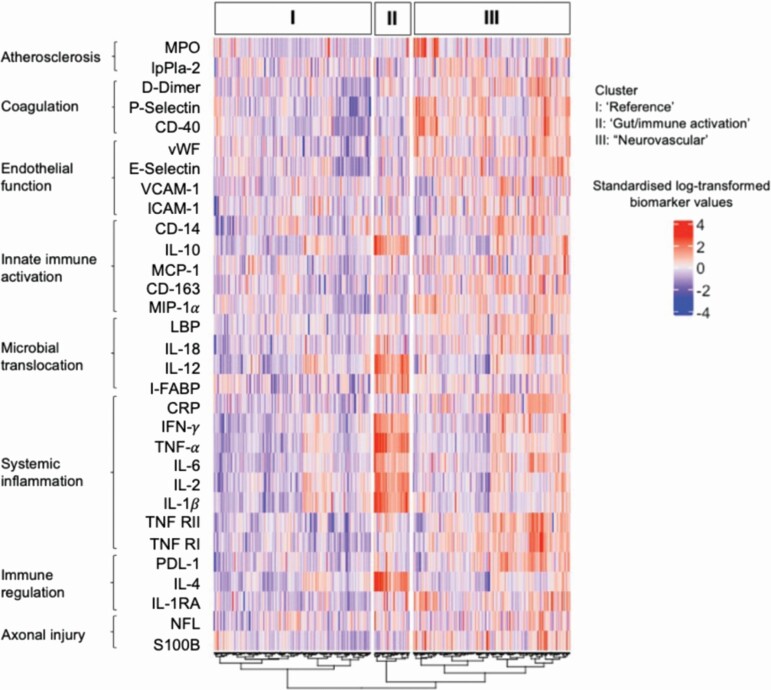
Heatmap of log-transformed standardized biomarker values for the three clusters identified.

PCA on the 31 log-transformed biomarkers resulted in three PCs being retained, which together explained 41.5% of the total variance between these variables; the Scree plot (Supplementary Figure S2) demonstrated a clear differentiation between the third and the fourth PC. AHCA conducted on the three PC scores yielded three clusters using the ASW method. Figure 1 presents a heatmap of the log-transformed standardized biomarker values displaying the three clusters identified that have distinct inflammatory patterns: a “gut/immune activation” cluster comprised of a small number of participants overall (*n* = 47, 10% of subjects) with an upregulation of cytokines and biomarkers associated with gut microbial translocation as well as regulation of responses to immune activation and proinflammatory cytokines; a “neurovascular” cluster (*n* = 209, 45% of subjects) including those with an upregulation in markers including coagulation, vascular as well as neuronal markers; and a third cluster (*n* = 209, 45% of subjects) including those with no distinct upregulation in inflammatory pathways; this group thus serves as the “reference” cluster for subsequent analyses.

Whilst participants in the three clusters generally had similar characteristics at baseline, there were statistically significant between-cluster differences for several characteristics (Table 1). Relative to the “gut/immune activation” and “reference” clusters, the “neurovascular” cluster included higher proportions of people with HIV (“gut/immune activation”: 59.6%; “neurovascular”: 78.0%; “reference”: 72.7%, *p* = .03), individuals with obesity (body mass index (BMI)*>*30 kg/m^2^; 21.3%, 24.3% and 10.6% for the “gut/immune activation”, “neurovascular” and “reference” clusters, respectively, *p* = .001) and those with previous CVD (27.7%, 53.1%, 39.2%, *p* = .001), mental health disorder (29.8%, 45.9%, 31.1%, *p* = .004) or arthritis of the knee or hip (8.5%, 16.3%, 7.7%, *p* = .02). Relative to the “neurovascular” and “reference” clusters, those in the “gut/immune activation” cluster had a higher median systolic blood pressure (135, 126, 126 mmHg, *p* = .002), albeit with median values remaining in the normal range. There were no between-cluster differences in the proportion that reported the use of sleep medication for insomnia at the POPPY-Sleep substudy visit (4.3%, 7.2%, 10.5%, *p* = .33). Furthermore, among the subgroup of those with HIV in each cluster, there were no between-cluster differences in the percentages of participants with HIV-RNA*<*50 copies/mL (88.9%, 90.2%, 94.7%, *p* = .20) or on ART (100.0%, 97.5%, 97.4%, *p* > .99) or in the median CD4+ T-cell count (654, 610, 605 cells/mm^3^, *p* = .93).

**Table 1.** Participant characteristics overall and by cluster

		Cluster			
*n (%) or median [Q1-Q3], unless otherwise noted*	Overall (*n* = 465)*	“Gut/immune activation” (*n* = 47)	“Neurovascular” (*n* = 209)	‘Reference (*n* = 209)	*p* ^†^
***Demographics***					
Age in years	54 [50–60]	56 [52–61]	55 [50–60]	54 [48–60]	.08
Male	374 (80.4%)	32 (68.1%)	170 (81.3%)	172 (82.3%)	.08
White	408 (87.7%)	41 (87.2%)	178 (85.2%)	189 (90.4%)	.26
Living with HIV	343 (73.8%)	28 (59.6%)	163 (78.0%)	152 (72.7%)	.03
***Anthropometric Measurements***					
Obese (BMI ≥30 kg/m^2^)	82 (17.8%)	10 (21.3%)	50 (24.3%)	22 (10.6%)	.001
Systolic Blood Pressure (mmHg)	126 [117–140]	135 [125–154]	126 [116–138]	126 [116–138]	.002
Diastolic Blood Pressure (mmHg)	79 [72–86]	82 [73–90]	79 [70–86]	78 [72–84]	.23
***Lifestyle Factors***					
MSM sexuality/route of HIV transmission	332 (71.4%)	30 (63.8%)	147 (70.3%)	155 (74.2%)	.33
Current alcohol use	384 (82.6%)	41 (87.2%)	168 (80.4%)	175 (83.7%)	.45
History of recreational drug use in past 6 months	107 (23.0%)	8 (17.0%)	55 (26.3%)	44 (21.1%)	.26
Ever injected drugs^FE^	32 (6.9%)	2 (4.3%)	16 (7.7%)	14 (6.7%)	.77
***Comorbidities***					
History of cancer	58 (12.5%)	6 (12.8%)	33 (15.8%)	19 (9.1%)	.12
History of any AIDS event	96 (20.6%)	8 (17.0%)	52 (24.9%)	36 (17.2%)	.13
History of any mental health disorder	175 (37.6%)	14 (29.8%)	96 (45.9%)	65 (31.1%)	.004
History of chest disease	173 (37.2%)	13 (27.7%)	87 (41.6%)	73 (34.9%)	.13
History of any thyroid disease^FE^	18 (3.9%)	1 (2.1%)	9 (4.3%)	8 (3.8%)	.94
History of renal problems^FE^	7 (1.5%)	1 (2.1%)	5 (2.4%)	1 (0.5%)	.21
History of any diabetes^FE^	33 (7.1%)	1 (2.1%)	21 (10.0%)	11 (5.3%)	.07
History of Hepatitis B Virus	69 (14.8%)	8 (17.0%)	29 (13.9%)	32 (15.3%)	.83
History of Hepatitis C Virus^FE^	29 (6.2%)	4 (8.5%)	14 (6.7%)	11 (5.3%)	.64
History of cardiovascular disease	206 (44.3%)	13 (27.7%)	111 (53.1%)	82 (39.2%)	.001
History of arthritis of knee or hip	54 (11.6%)	4 (8.5%)	34 (16.3%)	16 (7.7%)	.02
***Sleep Medication***					
Sleep medication use for insomnia^FE^	39 (8.4%)	2 (4.3%)	15 (7.2%)	22 (10.5%)	.33

^FE^Fisher’s exact test was conducted due to expected cell counts of less than 5.

^†^ *p*-value for between-cluster differences.

*Note, the following variables were missing data (number of participants missing data overall in parentheses): Obese (5), Systolic Blood Pressure (3), Diastolic Blood Pressure (3), Ever injected drugs (1).

### Sleep outcomes

Overall, 82 (18%) of the 449 participants with ISI data met criteria for insomnia (ISI *>*15). Most other (secondary) self-reported and objective sleep outcomes suggested generally good sleep quality in this study population (Table 2).

**Table 2.** Results of analyses for primary and secondary sleep outcomes

		Cluster			
*Odds ratio (OR (95% CI)) or median [Q1-Q3], unless otherwise noted*	Overall (*n* = 465)*	“Gut/immune activation” (*n* = 47)	“Neurovascular” (*n* = 209)	“Reference” (*n* = 209)	*p* ^†^
***PRIMARY SLEEP OUTCOME: Insomnia (ISI>15)***					
**Logistic Regression Results, *Estimated OR of Insomnia (95% CI)*** ^**^					
Unadjusted	--	1.10 (0.44, 2.47)	1.21 (0.73, 2.02)	**REF**	.76
Adjusted (HIV status, age, sex, race)	--	1.40 (0.54 3.32)	1.12 (0.66, 1.90)	**REF**	.75
***SECONDARY SLEEP OUTCOMES: PROMIS, Actigraphy and Oximetry***					
**PROMIS Questionnaire T-scores, *median [Q1-Q3]***					
Sleep-Related Impairment T-score	49.0 [44.0–56.0]	47.3 [43.6–52.9]	50.3 [43.6–57.2]	48.9 [43.6–54.0]	.17
Sleep Disturbance T-score	51.0 [46.0–57.0]	50.1 [45.5–58.0]	52.2 [45.5–57.3]	49.0 [44.2–56.3]	.25
**Actigraphy, *median [Q1-Q3]***					
Mean wake after sleep onset (WASO;mins)	53.0 [39.0–73.0]	54.0 [40.0–67.0]	57.0 [41.3–78.0]	49.0 [38.0–70.3]	.08
SD of wake after sleep onset (WASO;mins)	19.0 [13.0–28.0]	20.0 [13.0–24.0]	21.0 [14.0–30.0]	17.0 [12.0–26.0]	.01
Mean length of awakenings (mins)	3.0 [2.5–3.7]	3.1 [2.6–3.7]	3.3 [2.7–4.1]	2.8 [2.3–3.5]	<.001
SD of length awakenings (mins)	0.8 [0.6–1.2]	0.8 [05–1.3]	0.9 [0.7–1.4]	0.7 [0.5–1.0]	<.001
Mean number of awakenings	18.0 [14.0–23.0]	18.0 [14.0–22.0]	18 [13.0–22.0]	18.0 [14.0–24.0]	.42
SD of number of awakenings	5 [4.0–7.0]	5.0 [4.0–6.0]	5.0 [4.0–7.0]	5.0 [4.0–7.0]	.65
Mean sleep duration (mins)	423.0 [384.0–458.0]	428.0 [406.0–455.3]	423.5 [377.3–462.3]	421.0 [386.5–455.3]	.60
SD of sleep duration (mins)	54.0 [39.0–80.0]	46.0 [39.0–73.0]	60.0 [39.0–85.8]	52.0 [38.8–75.0]	.16
Mean sleep maintenance efficiency (%)	89.0 [85.1–91.6]	89.2 [86.9–91.9]	88.4 [83.6–91.4]	89.8 [85.6–91.8]	.10
SD of sleep maintenance efficiency (%)	3.5 [2.4–4.9]	3.3[2.3–4.5]	3.8 [2.5–5.3]	3.3[2.3–4.7]	.10
Mean sleep onset latency (mins)	7.0 [6.0–9.0]	7.0 [6.0–8.0]	7.0 [6.0–9.0]	7.0 [6.0–9.0]	.63
SD of sleep onset latency(mins)	3.0 [2.0–5.0]	4.0 [2.0–5.0]	3.0 [2.0–6.0]	3.0 [2.0–5.0]	.53
Mean movement index (%)	17.2 [13.7–21.6]	16.7 [14.2–19.9]	17.8 [13.7–22.8]	16.5 [13.6–20.4]	.15
SD of movement index (%)	3.4 [2.4–4.9]	3.4 [2.3–4.3]	3.7 [2.6–5.7]	3.3 [2.3–4.6]	.008
**Oximetry, *median [Q1***–***Q3]***					
Oxygen Desaturation Index (ODI) (4% Desaturation) (events per hour)	3.1 [1.5–6.4]	2.4 [1.4–7.5]	3.6 [1.5–6.7]	3.0 [1.5–6.0]	.50
Percentage of time with SpO2 below 90%	0.3 [0.0–2.5]	0.1 [0.0–1.6]	0.5 [0.0–4.7]	0.2 [0.0–1.7]	.009

^†^For between-cluster differences. Joint (likelihood ratio) test that the coefficients for the “Gut/immune activation” and “Neurovascular” clusters are both 0 for the logistic regression analysis.

*Note, the following variables were missing data (number of participants missing data overall in parentheses): Insomnia (16), PROMIS SRI T-score (7), PROMIS SD T-score (10), Mean WASO (14), SD of WASO (14), Mean length of awakenings (14), SD of length of awakenings (14), Mean number of awakenings (14), SD of number of awakenings (14), Mean sleep duration (14), SD of sleep duration (14), Mean sleep maintenance efficiency (14), SD of sleep maintenance efficiency (14), Mean sleep onset latency (14), SD of sleep onset latency (14), Mean movement index (14), SD of movement index (14), ODI(21),Percentage of time with SpO2 below 90% (21).

^**^Profile likelihood-based CIs are presented. Refer to the Supplementary Material for the results of the logistic regression analyses using the Firth bias adjustment.

**Figure 2.**
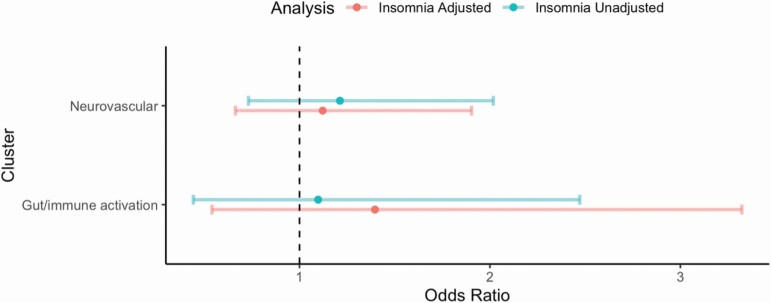
Estimated odds ratio (95% confidence interval) for insomnia comparing clusters (relative to the “reference” cluster; unadjusted and adjusted (HIV status, age, sex, race)).

There was no statistically significant between-cluster difference in the odds of insomnia in the “gut/immune activation” cluster (18.2% insomnia) relative to the “reference” cluster (16.8% insomnia) either before (crude odds ratio (cOR): 1.10 (95% CI: 0.44, 2.47)) or after adjustment for HIV status and other potential confounders (i.e. age, sex, race) (aOR: 1.40 (95% CI: 0.54, 3.32)). Similarly, there was no statistically significant between-cluster difference in the odds of insomnia in the “neurovascular” cluster (19.7% insomnia) relative to the “reference” cluster either before (cOR: 1.21 (95% CI: 0.73, 2.02)) or after adjustment for HIV status and other potential confounders (aOR: 1.12 (95% CI: 0.66, 1.90)) (Table 2 and Figure 2). LRTs testing for an overall association between insomnia and the clusters also indicated no statistically significant between-cluster differences, or association, either before (0.76) or after (0.75) adjustment. Sensitivity analyses are presented in the Supplementary Material; findings of these analyses were consistent with the results of the primary analyses.

Few associations were observed among the secondary objective and patient-reported sleep outcomes. Among most secondary sleep outcomes, the “gut/immune activation” and “neurovascular” clusters tended to exhibit more variable and disturbed sleep patterns in both patient-reported and objective actigraphy/oximetry sleep measures relative to the “reference” cluster, although most differences were not statistically significant or were considered to be clinically irrelevant (Table 2). Those in the “neurovascular” cluster spent a higher median percentage of time with SpO2 below 90% (“gut/immune activation”: 0.1%; “neurovascular”: 0.5%; “reference”: 0.2%, *p* = .009) relative to the other two clusters. Relative to the “reference” cluster, those in both the “gut/immune activation” and “neurovascular” clusters had higher medians of SD of WASO (20, 21, 17 min for the “gut/immune activation”, “neurovascular” and “reference” clusters, respectively, *p* = .01), mean length of awakenings (3.1, 3.3, 2.8 min, *p* < .001), SD of length of awakenings (0.8, 0.9, 0.7 min, *p* < .001), and SD of movement index (3.4%, 3.7%, 3.3%, *p* = .008).

Additional results of secondary sleep outcomes are presented in the Supplementary Material. We did not observe any marked differences between the results of the primary analyses and these secondary analyses, with the findings generally being consistent across all analyses.

## Discussion

Using data from a large study of people with HIV and appropriately-selected controls without HIV, we identified three clusters displaying distinct inflammatory profiles covering several inflammatory pathways similar to those identified in a previous smaller independent study in Ireland [37]. A “gut/immune activation” cluster whose members displayed a pattern of upregulation of cytokines and biomarkers associated with gut microbial translocation as well as regulation of responses to immune activation and proinflammatory cytokines; a “neurovascular” cluster whose members displayed a pattern of upregulation in markers including coagulation, vascular as well as neuronal markers; and a “reference” cluster whose members displayed a pattern of no distinct upregulation in inflammatory pathways relative to those in the other two clusters. Despite a numerically higher prevalence of insomnia among those in the “gut/immune activation” and “neurovascular” clusters than among those in the “reference” cluster, we did not find clinically or statistically significant associations between the inflammatory profile groups and insomnia either before or after adjustment for HIV status and other potential confounders. With the exception of a potentially interesting association with the percentage of time with SpO2 below 90%, few clinically relevant associations were observed among secondary actigraphy, oximetry, and PROMIS sleep outcomes.

Previous studies have reported associations of insomnia and sleep disturbances with elevated levels of inflammatory biomarkers, including several biomarkers considered in our analyses such as CRP, IL-6 and TNF-α [14, 15, 41]. Several studies focused on assessing the associations between inflammatory biomarkers and sleep problems in people with HIV have also been conducted [16, 19, 28, 30, 31], reporting associations between sleep disturbances and elevated levels of inflammation in this group. One previous study focused on the association between insomnia symptoms and biomarkers of monocyte activation, systemic inflammation, and coagulation in people with HIV. Whilst the study found only one statistically significant association between difficulty falling or staying asleep and higher levels of d-dimer, this association became non-significant after adjustment for demographic and clinical factors. A key limitation of this study was that it utilized the Veterans Aging Cohort Study (VACS) HIV Symptom Index to derive information on insomnia, a questionnaire that was not designed nor validated to screen for insomnia or its associated symptoms [19]. Another study reported associations between several inflammatory biomarkers and sleep initiation insomnia in people with HIV, determined using the Pittsburgh Sleep Quality Index. However, this questionnaire is designed to characterize global sleep quality rather than insomnia specifically, and the sample was a convenience sample from multiple health clinics and community sites in a single-area in the USA limiting the generalizability of the results [30]. Given the differing approaches in how insomnia and other sleep measures were assessed and sampling variability, it is difficult to directly compare our results with previous studies and elucidate why the “neurovascular” and “reference” clusters, both of which included higher proportions of people with HIV, did not experience higher levels of insomnia relative to the “gut/immune activation” cluster.

Based on previous work from the POPPY-Sleep sub-study, we also considered associations between the inflammatory profiles identified and several secondary patient-reported and objective sleep outcomes (PROMIS sleep questionnaires, actigraphy, and oximetry) [42]. We did not observe statistically significant or clinically meaningful between-cluster differences in the PROMIS-SRI or - SD T-scores, and we observed only a few associations with objective actigraphy-/oximetry-assessed measures that were statistically significant, but with only small magnitudes in these differences of unclear clinical significance. For example, we observed statistically significant between-cluster median differences in the percentage of time with SpO2 below 90%, an oximetry-assessed measure, which suggests that the “neurovascular” cluster includes more participants with a greater time with sleep-related hypoxia/hypoxemia (Table 2). This measure has been reported to be associated with an elevated inflammatory burden [43, 44], potentially providing some explanation for the relatively heightened inflammation in the “neurovascular” cluster. Although there is no consensus for the minimal clinically important difference in time with SpO2 below 90%, our observed absolute between-cluster median differences in the percentage of time with SpO2 below 90% were very small. From the actigraphy-assessed measures, there was strong evidence of between-cluster median differences in the SD of WASO, the mean and SD of length of awakenings, and SD of movement index; and although the between-cluster median differences of the other actigraphy-assessed measures were not statistically significant, they were consistent with the statistically significant actigraphy-assessed measures suggesting that those in the “gut/immune activation” and “neurovascular” clusters tend to experience greater sleep disturbance. Previous studies have reported associations between elevated levels of inflammation and actigraphy-assessed sleep disturbances, including longer sleep onset latency [45], shorter sleep duration [44] and sleep inconsistency in general covering actigraphy-assessed measures of WASO, sleep onset latency, terminal wakefulness, sleep duration/total time in bed and number of awakenings [14]. Although our findings for this present study do not support a strong association between inflammatory profiles and insomnia, we do report associations between inflammatory profiles and several secondary objective sleep outcomes. Specifically, we report statistically significant associations between inflammatory profiles and the mean length of awakenings and variability in three objective measures of sleep disruption: length of awakenings, WASO, and movement index among actigraphy-assessed measures; as well as percentage of time with SpO2 below 90% among oximetry-assessed measures. While differences are small, they point to sleep measures that may relate to inflammation and are consistent with a growing literature indicating that not only averaged sleep metrics, but measures of night-to-night variability in sleep, associate with adverse outcomes, including metabolic dysfunction, potentially reflecting effects of circadian disruption [46]. In addition, it is possible associations may exist with other sleep outcomes not explored in this study.

The strengths of our study include: (1) our large sample of people currently living with HIV and appropriately-selected HIV-negative controls from multiple health centers in England and Ireland that were recruited into the POPPY-Sleep sub-study regardless of sleep problems, (2) our use of validated instruments to classify insomnia symptoms, and (3) assessment of both patient-reported and objective sleep measures. Also, we now provide external validation of biomarker clusters identified in a previous smaller independent study [37], and to our knowledge, this is the first study to explore associations between inflammatory profiles identified in a sample of people with HIV and HIV-negative controls and insomnia as well as secondary patient-reported and objective sleep measures.

Nevertheless, there are several limitations to our study. First, although the sample size overall is larger than previous studies exploring associations between inflammation and insomnia and/or other sleep disturbances in people with HIV, it may not be sufficiently large to detect between-cluster differences in all sleep measures explored in this study, particularly given the relatively small sample size of the “gut/immune activation” cluster. Additionally, the small number of HIV-negative controls reporting insomnia (*n* = 6; 1, 3 and 2 in the “gut/immune activation”, “neurovascular” and “reference” clusters, respectively) and the relatively small number of participants with insomnia overall (*n* = 82) meant that we were limited in the number of potential confounders that could be reasonably included in our multivariable models. However, results from sensitivity analyses with Firth bias adjustment were comparable to our main results, suggesting that any bias introduced is likely to be minimal. Also, whilst we have considered many potential confounders, our results may be affected by residual confounding because of our inability to fully adjust for some other observed (e.g. employment status and alcohol use) and unobserved (e.g. physical activity status, and use of anti-inflammatory and/or sleep medications from comprehensive medical prescription records) confounders. This, together with the cross-sectional nature of our analysis, limits our ability to demonstrate causal associations. However, conclusions did not change after additional adjustment for self-reported current alcohol use and employment status and sleep medication use for insomnia, all as binary covariates, and thus we do not expect substantial changes in our conclusions if more detailed information was available on these measures (Supplementary Material). It is important to acknowledge that the self-reported sleep medication use data are subject to recall bias. Finally, whilst we included a broad range of biomarkers covering different biological pathways, we can’t rule out the possibility that other unmeasured biomarkers may be more strongly associated with sleep outcomes in this group.

We should also note that the three PCs retained after log-transformation for the AHCA accounted for only 41.5% of the total variance among all 31 log-transformed biomarker variables, and this may have resulted in a loss of information. However, retaining additional PCs may have identified clusters that reflect uninformative random variation rather than meaningful true variation in the 31 biomarker measurements, and the approach taken identified a third cluster that was potentially masked by the variation in the raw data, where AHCA identified only two clusters. Also, the results of analyzing each biomarker separately were consistent with the analyses using the clusters identified, suggesting minimal loss of information.

We recognize that the inclusion of HIV-negative controls and/or those people living with HIV that experienced viraemia at the POPPY baseline visit may have introduced population stratification/heterogeneity bias. However, PCA is commonly used in genome-wide association studies to reduce population stratification/heterogeneity bias, and as such would be expected to lead to a reduction in any bias introduced [47]. Results from our sensitivity analyses suggested that the inflammatory clusters identified are robust to the exclusion of HIV-negative controls or those people living with HIV that experienced viraemia at the POPPY baseline visit, with all analyses yielding three clusters that were visually very similar (Supplementary Material). However, we do observe an amplified adjusted odds ratio for the “neurovascular” cluster associated with relatively elevated levels of biomarkers across a range of inflammatory pathways in the analysis which excluded HIV-negative controls compared to the primary analysis (odds ratios: 1.62 (95% CI: 0.98, 2.89) in sensitivity analyses vs. 1.12 (95% CI: 0.66, 1.90) in primary analyses); similar changes in the point estimates are observed in the linear regression analysis considering the ISI score as a continuous outcome (point estimates: 1.28 (95% CI: −0.36, 2.92) vs. 0.76 (95% CI: −0.41, 1.93)). Although the lack of statistical significance of these findings remains unchanged, these results may justify further investigation into the association between inflammatory biomarker clusters and sleep outcomes in larger cohorts. Additionally, inclusion of these individuals further generalizes results to patient populations that are likely to present to care in non-HIV care settings. Whilst a small number of participants lacked data on our primary outcome, sensitivity analyses on missing data for insomnia yielded similar results to the complete case analysis. Last, we must recognize issues of data multiplicity and type I error inflation in the analyses of sleep outcomes and baseline characteristics. Our analyses did not formally adjust for multiple testing, as many of the outcomes are not independent, but are likely positively- and negatively-dependent. Adjustment using methods such as Bonferroni correction would be inappropriate for this situation, and the Benjamini and Yakutieli method that allows for positively- and negatively-dependent statistical tests tends to lack statistical power or is very conservative [48]. Insomnia was selected (*a priori*) as the primary outcome of interest, with the secondary sleep outcomes included mainly for confirmatory purposes. As such, we placed less emphasis on statistical significance for these associations.

## Conclusions

We did not find any clinically meaningful or strong direct associations between inflammatory profiles and insomnia as well as a wide range of secondary sleep outcomes. Although associations could exist with other sleep outcomes (e.g. sleep architecture) or biomarker types (e.g. cerebrospinal fluid) not assessed, our findings do not support a strong association between sleep quality/patterns and inflammatory profiles in this population. Further research is needed to understand the inflammatory pathways that may affect the pathogenesis of insomnia, sleep-related hypoxia/hypoxemia and other sleep disorders in people with HIV not elucidated by our study.

## Supplementary Material

zsac212_suppl_Supplementary_Material

## Data Availability

Authors are unable to share the data in compliance of the consent agreement signed by all study participants.
