# Supplementary material for: Biomarker associations with insomnia and secondary sleep outcomes in persons with and without HIV in the POPPY-Sleep substudy: a cohort study
Source: Sleep. 2022 Sep 14;45(12):zsac212. doi: 10.1093/sleep/zsac212 (PMC9742892; doi:10.1093/sleep/zsac212)
Supplement: zsac212_suppl_Supplementary_Material [file zsac212_suppl_supplementary_material.pdf]

Biomarker Associations with Insomnia and Secondary Sleep Outcomes in Persons with and without HIV in the POPPY-Sleep Sub-study: a cohort study

Supplementary Material

Nicholas Bakewell[1], Caroline A Sabin[1], Riya Negi[2], Alejandro Garcia-Leon[2], Alan Winston[3], Memory Sachikonye[4], Nicki Doyle[3], Susan Redline[5,6,7], Patrick WG Mallon[2*], Ken M Kunisaki[8,9*]

[1]Institute for Global Health, University College London, London, UK; [2]Centre for Experimental Pathogen Host Research, School of Medicine, University College Dublin; [3]Department of Infectious Disease, Imperial College London, London, UK; [4]UK Community Advisory Board (UK-CAB), London, UK; [5]Brigham and Women's Hospital, Boston, USA; [6]Harvard Medical School, Harvard University, Boston, USA; [7]Beth Israel Deaconess Medical Center, Boston, USA; [8]Minneapolis Veterans Affairs Health Care System, Minneapolis, USA; [9]University of Minnesota, Minneapolis, USA

[*]PWGM and KMK are joint senior authors.

Corresponding author: Caroline Sabin (c.sabin@ucl.ac.uk)

Section 1: Additional Details of Principal Components Analysis for Primary Outcome

Figure S1. PC loadings of the first three PCs retained (large loadings in red, dashed line represents the threshold for large loadings)

Figure S2. Scree plot from the PCA on the 31 log-transformed biomarker variables

Figure S3. Cluster analysis dendrogram

Figure S4. 3-Dimensional plot of the three PC scores of participants, colour-coded by cluster

Section 2: Assessment of Model Fit and Sensitivity Analyses

Section 3: Supplemental results

Table S1. Supplementary results of logistic regression analyses with Firth bias adjustment (top) and secondary sleep outcomes (bottom)

Figure S5. Estimated odds ratio (95% confidence interval) for insomnia comparing clusters (relative to the 'reference' cluster; unadjusted and adjusted (HIV status, age, sex, race)), Firth bias adjustment

Table S2. Missing data sensitivity analyses for the primary insomnia logistic regression analyses (no Firth bias adjustment)

Analyses of insomnia severity index as a continuous outcome, excluding HIV-negative controls, and excluding HIV-viraemic participants

Figure S6. Heatmap of log-transformed standardised biomarker values for the three clusters identified excluding HIV-negative controls (dendrogram at bottom of heatmap)

Figure S7. Heatmap of log-transformed standardised biomarker values for the three clusters identified excluding people living with HIV that experienced viraemia (HIV-RNA>50 copies/mL) at the POPPY baseline visit (dendrogram at bottom of heatmap)

Table S3. Participant characteristics overall and by cluster (excluding HIV-negative controls)

Table S4. Participant characteristics overall and by cluster (excluding people living with HIV that experienced viraemia (HIV-RNA>50 copies/mL) at the POPPY baseline visit)

Table S5. Unadjusted and adjusted logistic regression (Insomnia, ISI ≥15) and linear regression (ISI score, continuous) results for (a) analysis excluding HIV-negative controls and (b) analysis excluding people living with HIV that experienced viraemia (HIV-RNA>50 copies/mL) at the POPPY baseline visit

Table S6. Logistic regression (Insomnia, ISI ≥15) and linear regression (ISI score, continuous) results for all 31 individual (log-transformed) biomarkers: unadjusted and adjusted (HIV status, age, sex, race)

Section 1: Principal Component Analysis Results for Primary Analysis

Figure S1 presents the loadings for the three principal components (PCs) retained, with large loadings in red. Large loadings are defined as loadings with an absolute value greater than or equal to $\sqrt{1/31}$, since it is expected that the squared loadings are equal to $1/31$ if all biomarkers contributed equally to the variance of a PC. Dashed line represents the threshold for large loadings).

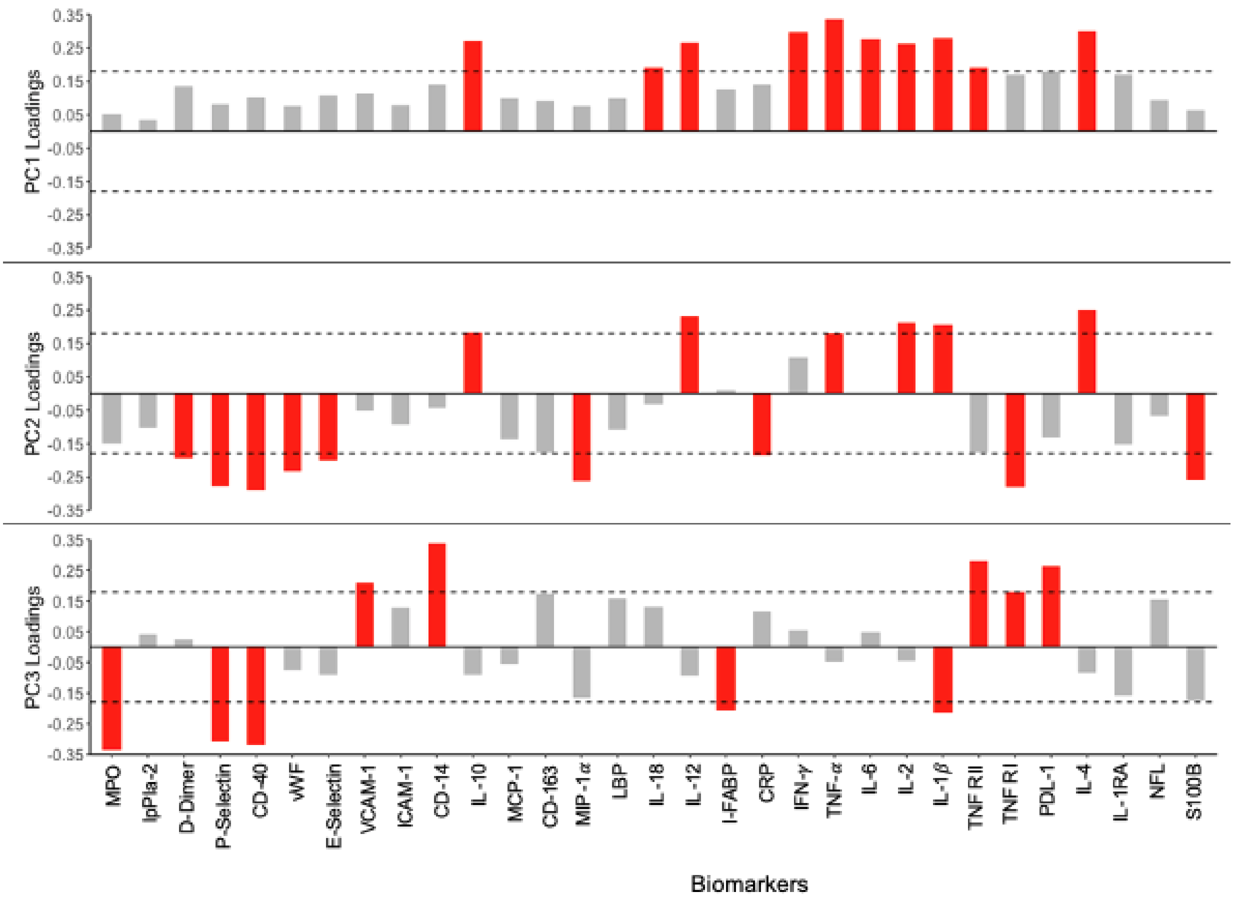

Figure S2 presents the Scree plot from the Principal Component Analysis (PCA) of the 31 log-transformed biomarkers, where the elbow occurs at the third PC.

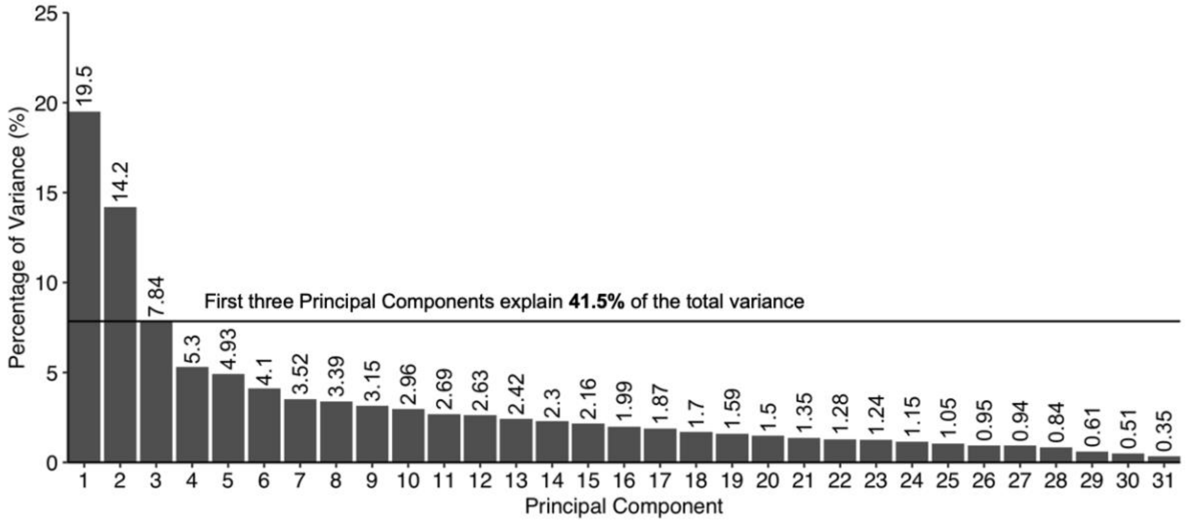

Figure S3 presents the dendrogram of the agglomerative hierarchical cluster analysis (AHCA) with outlines around the three clusters identified using the average silhouette width (ASW) method; most other methods for determining the optimal number of clusters also identified three clusters.

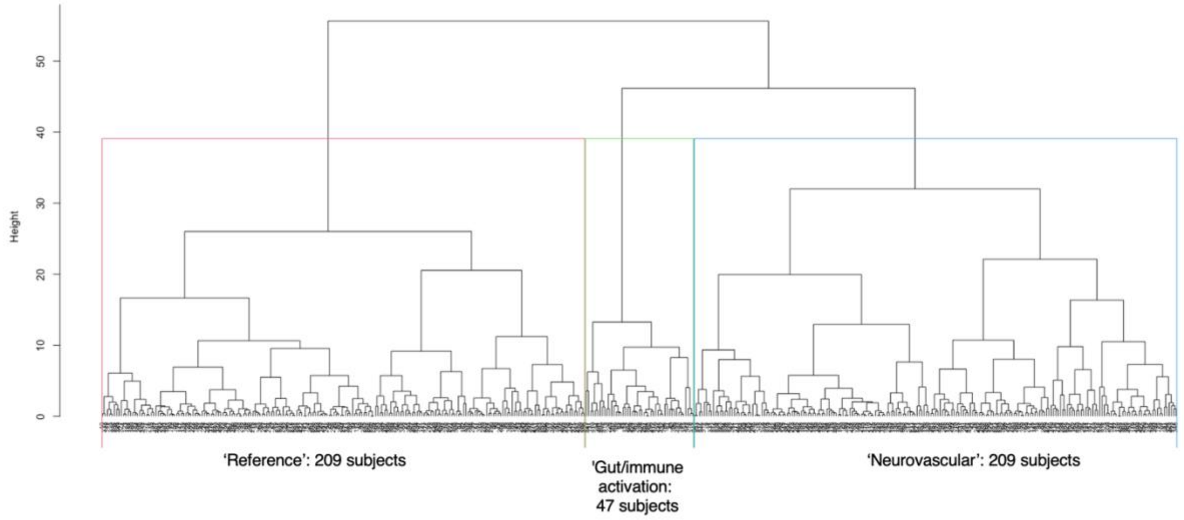

Figure S4 displays a 3-Dimensional plot of the participants using coordinates based on their three PC scores to graphically demonstrate the separation of the three clusters identified.

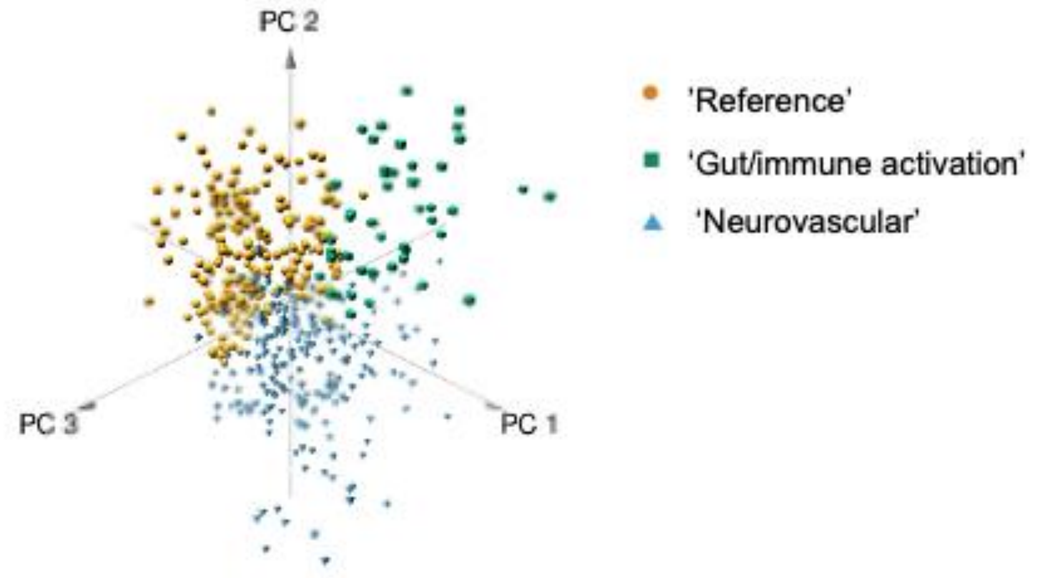

Section 2: Assessment of Model Fit and Sensitivity Analyses

Methods

Model fit of the logistic regression models was assessed using the Hosmer-Lemeshow test and examining Pearson residuals. Models were refitted with Firth bias adjustment to assess the sensitivity of the results to potential sparse data bias. Current employment status and alcohol use, and sleep medication use for insomnia (collected at the POPPY-Sleep sub-study visit) were added to the adjusted (HIV status, age, sex, race) model refitted with Firth bias adjustment. This further adjustment was done to assess the impact of excluding potential confounders we have collected data on, even though a limited set of confounders was selected in the primary analysis to minimise potential sparse data bias. Sensitivity analyses were also conducted on missing data for the insomnia analysis, assuming all participants missing data on insomnia either had insomnia or did not. There were no missing data in the covariates included in the primary adjusted logistic regression analysis (HIV status, age, sex, race).

Furthermore, we conducted a sensitivity analysis considering the Insomnia Severity Index (ISI) score as a continuous outcome and fitting linear regression models for the unadjusted and adjusted (HIV status, age, sex, race) analyses. We also conducted sensitivity analyses re-running the PCA and AHCA to assess the sensitivity and stability of the clusters identified when excluding HIV-negative controls or those people living with HIV experiencing viraemia (HIV-RNA>50 copies/mL) at the POPPY baseline visit. Here, we only describe the clusters based on the heatmaps generated and participant characteristics, and do not provide clinically-relevant labels for the clusters identified to avoid confusion with the primary analysis that included all POPPY-Sleep sub-study participants with reliable biomarker values. We also provide results for the primary outcome analyses using the clusters identified in these analyses (i.e. unadjusted and adjusted (HIV status, age, sex, race) analyses for both the binary insomnia (ISI ≥15) and continuous ISI score outcomes). Lastly, the individual biomarker associations with insomnia (ISI ≥15) and the continuous ISI score were explored using logistic regression and linear regression, respectively. The biomarker values were (natural) log-transformed prior to analysis; and unadjusted and adjusted results (HIV status, age, sex, race) analyses were conducted to align with the primary analysis.

Results/Discussion

The Hosmer-Lemeshow test indicated poor model fit for the unadjusted analysis ($p<0.001$, assuming 5 groups), however, there was no evidence to reject the null hypothesis that the model was correctly specified after adjustment (HIV status, age, sex, race; $p=0.86$, assuming 5 groups). Conclusions were consistent with results of Hosmer-Lemeshow test assuming several different values for the number of groups. The Pearson residuals in the unadjusted model also indicated poor model fit, with low fitted probabilities below 0.5 for all participants suggesting poor calibration and discrimination. Similarly, although the Hosmer-Lemeshow resulted in no evidence to reject the null hypothesis that the model is correctly specified in the adjusted analysis, this is not evidence that the model is correctly specified and the Pearson residuals indicated that the model fit was not optimal, with notably high positive outlying residuals for participants who were older, HIV-negative and white.

The results of logistic regression analyses were similar after Firth bias adjustment before and after adjustment for potential confounders (**Table S1** and **Figure S5**), with slightly larger point estimates and wider confidence intervals (CIs) for the 'gut/immune activation' cluster in both the unadjusted and adjusted analyses, and nearly identical point estimates and CIs for the 'neurovascular' cluster in both the unadjusted and adjusted analyses. The conclusions drawn from the adjusted model including current employment status and alcohol use and sleep medication use for insomnia were consistent with the results of the primary analysis adjusted model excluding these variables; however, the point estimates did vary between the adjusted

analyses. This may be due to the missingness in current employment status that led to the exclusion of participants with insomnia (n=7; 0, 5 and 2 in the ‘gut/immune activation’, ‘neurovascular’ and ‘reference’ clusters, respectively), and 32 participants overall (n=1, 16 and 15 in the ‘gut/immune activation’, ‘neurovascular’ and ‘reference’ clusters, respectively), potentially introducing further sparse data bias in the logistic regression analysis (**Table S1**).

Section 3: Supplementary Results

Table S1. Supplementary results of logistic regression analyses with Firth bias adjustment (top) and secondary sleep outcomes (bottom)

Odds ratio (OR) (95% confidence interval (CI)) or median [Q1-Q3], unless otherwise noted	**Overall (n=465)***	**Cluster**			**p†**
		**'Gut/immune activation' (n=47)**	**'Neurovascular' (n=209)**	**'Reference' (n=209)**	
PRIMARY SLEEP OUTCOME: Insomnia (ISI≥15)					
**Insomnia Logistic Regression Results with Firth Bias Adjustment,** ***Estimated OR of Insomnia (95% CI)****					
Unadjusted	--	1.14 (0.47, 2.52)	1.21 (0.73, 2.01)	**REF**	0.76
Adjusted (HIV status, age, sex, race)	--	1.43 (0.57, 3.34)	1.12 (0.67, 1.89)	**REF**	0.71
Adjusted (HIV status, age, sex, race, current alcohol use, current employment status, sleep medication use for insomnia)	--	1.73 (0.67, 4.17)	1.05 (0.59, 1.85)	**REF**	0.50
SECONDARY SLEEP OUTCOMES: Actigraphy and Oximetry					
**Actigraphy*****, median [Q1-Q3]***					
Average fragmentation index (%)	12.6 [9.3-16.0]	13.0 [9.8-15.3]	12.9 [9.7-16.2]	12.1 [9.0-16.0]	0.65
SD of fragmentation index (%)	8.0 [6.3-10.0]	7.4 [6.6-9.5]	8.3 [6.3-10.3]	7.8 [6.3-9.7]	0.48
Average in-bed duration per main sleep (mins)	485.0 [447.0- 526.0]	486.0 [460.0-528.0]	487.5 [443.0-533.8]	483.0 [450.0-515.3]	0.52
SD of in-bed duration per main sleep (mins)	64.0 [44.0-90.0]	56.0 [44.0-85.0]	68.5 [45.3-98.0]	58.5 [42.0-85.0]	0.05
Average sleep period duration per main sleep (mins)	477.0 [440.5- 518.0]	477.0 [454.0- 521.0]	480.0 [436.0-525.0]	477.0 [441.0-508.3]	0.58
SD of sleep period duration per main sleep (mins)	63.0 [43.0-91.0]	57.0 [44.0-88.0]	67.0 [46.3-97.5]	58.5 [42.8-84.3]	0.05
**Oximetry,** ***median [Q1-Q3]***					
Oxygen Desaturation Index (ODI) (4% Desaturation) (events per hour) adjusted	3.5 [1.7-7.1]	3.0 [1.6-9.6]	4.2 [2.0-7.7]	3.2 [1.7-6.4]	0.13

†For between-cluster differences; joint (likelihood ratio) test that the coefficients for the 'Gut/immune activation' and 'Neurovascular' clusters are both 0 for the logistic regression analysis.
*Note, the following variables were missing data (number of participants missing data overall in parentheses): Insomnia (16), Current employment status (32; 2 missing insomnia), Average fragmentation index (14), SD of fragmentation index (14), Average in-bed duration per main sleep (14), SD of in-bed duration per main sleep (14), Average sleep period duration per main sleep (14), SD of sleep period duration per main sleep (14), ODI adjusted (21).
**Profile likelihood-based CIs are presented.

Figure S5. Estimated odds ratio (95% confidence interval) for insomnia comparing clusters (relative to the ‘reference’ cluster; unadjusted and adjusted (HIV status, age, sex, race), Firth bias adjustment.

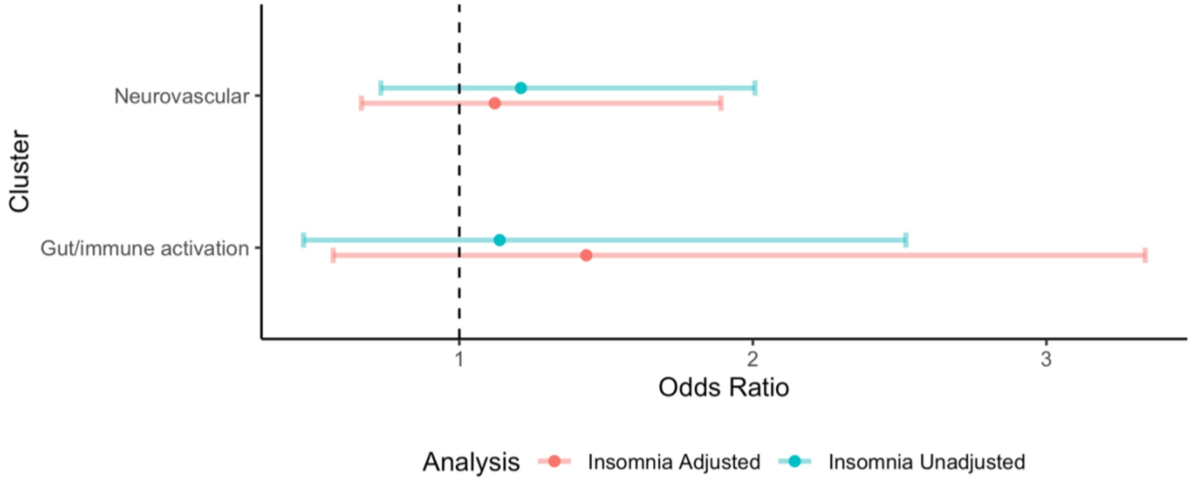

Table S2. Missing data sensitivity analyses for the primary insomnia logistic regression analysis (no Firth bias adjustment). This analysis was conducted assuming all participants missing insomnia data either have insomnia (top) or do not (bottom) resulted in similar conclusions to the complete case analysis, with no statistically significant associations between cluster membership and insomnia before or after adjustment for potential confounders. However, the point estimates and CIs did differ, likely because a relatively large proportion of participants missing outcome data were either in the 'reference' (7 [44%] of the participants missing outcome data) or 'neurovascular' (6 [38%] of the participants missing outcome data) clusters, while only 3 participants (19% of the participants missing outcome data) were missing insomnia data in the 'gut/immune activation' cluster.

Odds ratio (OR) (95% confidence interval (CI)), unless otherwise noted	**Cluster**			**p†**
	**'Gut/immune activation' (n=47)**	**'Neurovascular' (n=209)**	**'Reference' (n=209)**	
**Assuming all participants missing outcome data have insomnia (ISI≥15): Insomnia Logistic Regression Results, *Estimated OR of Insomnia (95% CI)*****				
Unadjusted	1.25 (0.57, 2.60)	1.16 (0.72, 1.86)	**REF**	0.77
Adjusted (HIV status, age, sex, race)	1.49 (0.64, 3.28)	1.02 (0.62, 1.68)	**REF**	0.63
**Assuming all participants missing outcome data do not have insomnia (ISI<15): Insomnia Logistic Regression Results, *Estimated OR of Insomnia (95% CI)*****				
Unadjusted	1.06 (0.43, 2.36)	1.22 (0.74, 2.02)	**REF**	0.74
Adjusted (HIV status, age, sex, race)	1.30 (0.51, 3.06)	1.14 (0.68, 1.93)	**REF**	0.80

†For between-cluster differences; joint (likelihood ratio) test that the coefficients for the 'Gut/immune activation' and 'Neurovascular' clusters are both 0 for the logistic regression analysis.
**Profile likelihood-based CIs are presented.

Insomnia Severity Index (ISI) as a continuous outcome

Considering the ISI score as a continuous outcome, there was no statistically significant between-cluster difference in the mean ISI score in the ‘gut/immune activation’ cluster (mean (standard deviation) ISI score: 8.14 (5.96)) relative to the ‘reference’ cluster (mean ISI score: 7.94 (6.08)) either before (estimated crude mean difference: 0.20 (95% CI: -1.79, 2.18)) or after adjustment (HIV status, age, sex, race; estimated adjusted mean difference: 0.87 (95% CI: -0.32, 2.05)). Similarly, there was no statistically significant between-cluster difference in the mean ISI score in the ‘neurovascular’ cluster (mean ISI (SD): 8.81 (6.09)) relative to the ‘reference’ cluster either before (estimated crude mean difference: 0.57 (95% CI: -1.38, 2.52)) or after adjustment (HIV status, age, sex, race; estimated adjusted mean difference: 0.76 (95% CI: -0.41, 1.93)). Likelihood ratio tests for an overall association between ISI scores and the clusters also indicated no statistically significant between-cluster differences, or association, either before (p=0.35) or after (p=0.43) adjustment.

Analyses excluding HIV-negative controls and HIV-viraemic participants

For the analysis that excluded HIV-negative controls, PCA on the 31 log-transformed biomarkers resulted in three PCs being retained, which together explained 40.9% of the total variance between these variables. Similar results were observed for the analysis that excluded HIV-viraemic participants (at the POPPY baseline visit), with three PCs being retained, which together explained 42.0% of the total variance between these variables. In both these sensitivity analyses, three clusters were identified using the ASW method (**Figures S6** and **S7**). For the analysis that excluded HIV-negative controls, Cluster I (n=216) included individuals with relatively low levels of inflammation; Cluster II (n=39) included those with relatively high levels of markers associated with systemic inflammation, immune regulation microbial translocation and innate immune activation; and Cluster III (n=88) included those with relatively high levels of inflammation across a range of inflammatory pathways. For the analysis that excluded HIV-viraemic participants, the three clusters identified were visually similar to those previously described for the analysis that excluded HIV-negative controls, which were also visually very similar to the clusters identified using the whole study population, but had different cluster sizes: Cluster I (n=295), Cluster II (n=58), and Cluster III (n=83).

Figure S6. Heatmap of log-transformed standardised biomarker values for the three clusters identified excluding HIV-negative controls (cluster analysis dendrogram at bottom of heatmap)

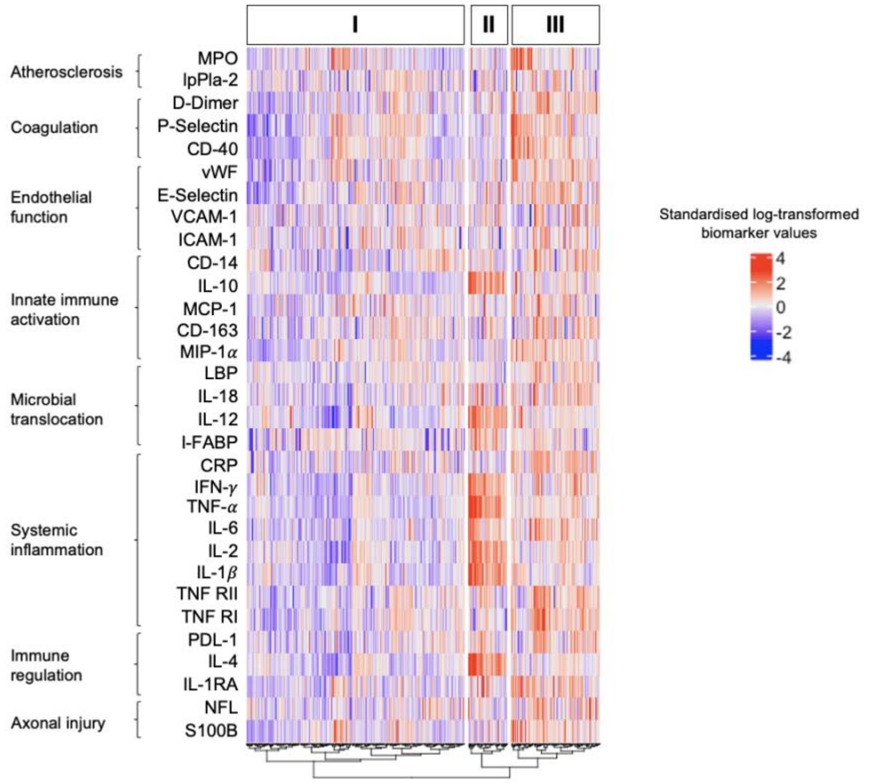

Figure S7. Heatmap of log-transformed standardised biomarker values for the three clusters identified excluding people living with HIV that experienced viraemia (HIV-RNA>50 copies/mL) at the POPPY baseline visit (cluster analysis dendrogram at bottom of heatmap)

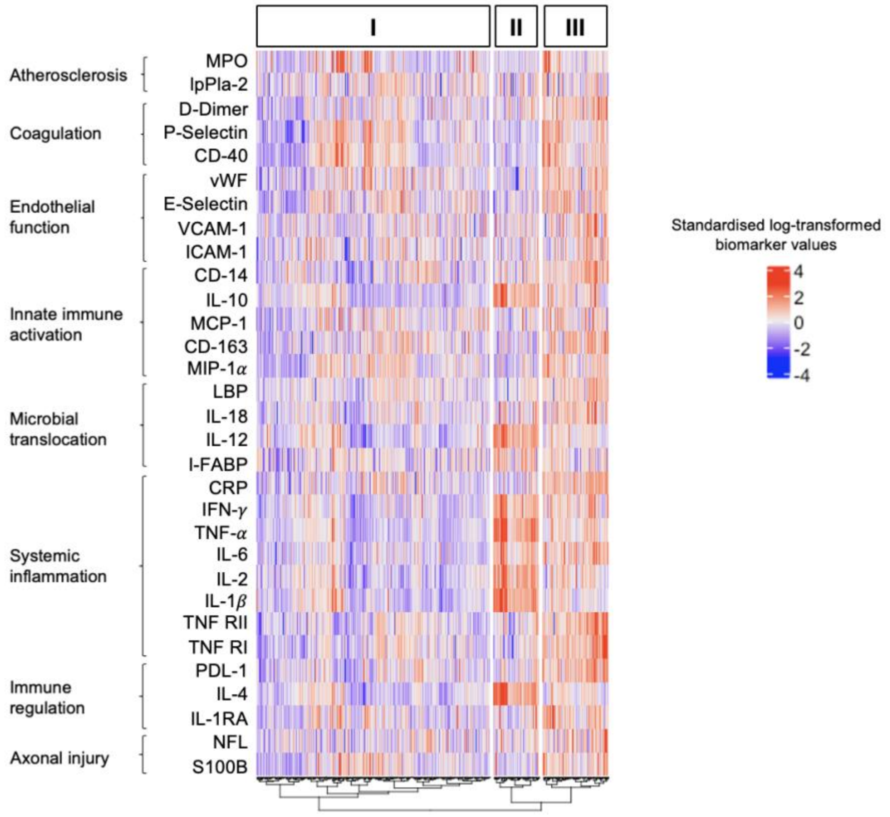

The between-cluster differences in participant characteristics were generally similar to those observed in the primary analysis, however, there were some notable differences. For the analysis that excluded HIV-negative controls, there were between-cluster differences in median age, median diastolic blood pressure, and proportion with renal problems that were not observed in the primary analysis (**Table S3**, below). For the analysis that excluded HIV-viraemic participants at the POPPY baseline visit, there were between-cluster differences in median age, and proportions with previous cancer, chest disease, renal problems and diabetes that were not observed in the primary analysis (**Table S4**). Additionally, we no longer observed statistically significant between-cluster differences in the proportions with previous mental health disorders and arthritis of the knee or hip. For the analyses on the outcomes, we observed consistent results for the logistic regression analysis (insomnia, ISI ≥15) and the linear regression analysis using the ISI score (continuous) across all three PCA-AHCA analyses, with no statistically or clinically significant differences observed before or after adjustment (**Table S5**).

Table S3. Participant characteristics overall and by cluster (excluding HIV-negative controls)

n (%) or median [Q1-Q3], unless otherwise noted	Overall (n=343)*	Cluster			p†
		I (n=216)	II (n=39)	III (n=88)	
Demographics					
Age in years	53 [47-59]	51 [46-57]	53 [50-60]	55 [50-59]	0.01
Male	292 (85.1%)	185 (85.6%)	31 (79.5%)	76 (86.4%)	0.57
White	299 (87.2%)	189 (87.5%)	34 (87.2%)	76 (86.4%)	0.96
Anthropometric Measurements					
Obese (BMI ≥30 kg/m^2)	56 (16.4%)	26 (12.1%)	10 (25.6%)	20 (23.0%)	0.02
Systolic Blood Pressure (mmHg)	126 [117-137]	124 [116-134]	138 [126-154]	128 [117-138]	<0.001
Diastolic Blood Pressure (mmHg)	79 [72-86]	78 [72-85]	84 [76-92]	78 [70-86]	0.04
Lifestyle Factors					
MSM sexuality/route of HIV transmission	269 (78.4%)	172 (79.6%)	30 (76.9%)	67 (76.1%)	0.78
Current alcohol use	273 (79.6%)	175 (81.0%)	32 (82.1%)	66 (75.0%)	0.46
History of recreational drug use in past 6 months	90 (26.2%)	56 (25.9%)	7 (17.9%)	27 (30.7%)	0.32
Ever injected drugs[FE]	30 (8.8%)	15 (7.0%)	3 (7.7%)	12 (13.6%)	0.17
Comorbidities					
History of cancer	47 (13.7%)	26 (12.0%)	5 (12.8%)	16 (18.2%)	0.36
History of any AIDS event	96 (28.0%)	54 (25.0%)	11 (28.2%)	31 (35.2%)	0.20
History of any mental health disorder	144 (42.0%)	89 (41.2%)	14 (35.9%)	41 (46.6%)	0.49
History of chest disease	144 (42.0%)	86 (39.8%)	13 (33.3%)	45 (51.1%)	0.10
History of any thyroid disease[FE]	10 (2.9%)	6 (2.8%)	0 (0.0%)	4 (4.5%)	0.44

Table S3. Participant characteristics overall and by cluster (excluding HIV-negative controls)

History of renal problems[FE]	6 (1.7%)	1 (0.5%)	1 (2.6%)	4 (4.5%)	0.04
History of any diabetes[FE]	24 (7.0%)	12 (5.6%)	2 (5.1%)	10 (11.4%)	0.20
History of Hepatitis B Virus	62 (18.1%)	35 (16.2%)	12 (30.8%)	15 (17.0%)	0.09
History of Hepatitis C Virus[FE]	28 (8.2%)	17 (7.9%)	5 (12.8%)	6 (6.8%)	0.53
History of cardiovascular disease	161 (46.9%)	95 (44.0%)	15 (38.5%)	51 (58.0%)	0.05
History of arthritis of knee or hip[FE]	37 (10.8%)	21 (9.7%)	4 (10.3%)	12 (13.6%)	0.63
Sleep Medication					
Sleep medication use for insomnia[FE]	36 (10.5%)	24 (11.1%)	3 (7.7%)	9 (10.2%)	0.90

[FE]Fisher's exact test was conducted due to expected cell counts of less than 5.
†p-value for between-cluster differences.
*Note, the following variables were missing data (number of participants missing data overall in parentheses): Obese (2), Systolic Blood Pressure (2), Diastolic Blood Pressure (2), Ever injected drugs (1).

Table S4. Participant characteristics overall and by cluster (excluding people living with HIV that experienced viraemia (HIV-RNA>50 copies/mL) at the POPPY baseline visit)

n (%) or median [Q1-Q3], unless otherwise noted	Overall (n=436)*	Cluster			p†
		I (n=295)	II (n=58)	III (n=83)	
Demographics					
Age in years	55 [50-60]	54 [48-59]	57 [52-61]	57 [52-64]	<0.001
Male	353 (81.0%)	236 (80.0%)	45 (77.6%)	72 (86.7%)	0.30
White	385 (88.3%)	256 (86.8%)	55 (94.8%)	74 (89.2%)	0.21
Living With HIV	314 (72.0%)	214 (72.5%)	32 (55.2%)	68 (81.9%)	0.002
Anthropometric Measurements					
Obese (BMI ≥30 kg/m^2)	74 (17.2%)	48 (16.4%)	6 (10.3%)	20 (24.7%)	0.07
Systolic Blood Pressure (mmHg)	126 [117-140]	126 [116-137]	134 [124-149]	129 [118-140]	0.004
Diastolic Blood Pressure (mmHg)	79 [72-86]	78 [71-84]	81 [74-90]	80 [74-87]	0.19
Lifestyle Factors					
MSM sexuality/route of HIV transmission	314 (72.0%)	209 (70.8%)	41 (70.7%)	64 (77.1%)	0.52
Current alcohol use	361 (82.8%)	247 (83.7%)	49 (84.5%)	65 (78.3%)	0.48
History of recreational drug use in past 6 months	99 (22.7%)	67 (22.7%)	11 (19.0%)	21 (25.3%)	0.68
Ever injected drugs[FE]	28 (6.4%)	20 (6.8%)	3 (5.2%)	5 (6.0%)	>0.99
Comorbidities					
History of cancer	54 (12.4%)	30 (10.2%)	7 (12.1%)	17 (20.5%)	0.04
History of any AIDS event	94 (21.6%)	61 (20.7%)	10 (17.2%)	23 (27.7%)	0.27
History of any mental health disorder	162 (37.2%)	104 (35.3%)	21 (36.2%)	37 (44.6%)	0.30

Table S4. Participant characteristics overall and by cluster (excluding people living with HIV that experienced viraemia (HIV-RNA>50 copies/mL) at the POPPY baseline visit)

History of chest disease	165 (37.8%)	106 (35.9%)	15 (25.9%)	44 (53.0%)	0.002
History of any thyroid disease[FE]	17 (3.9%)	13 (4.4%)	1 (1.7%)	3 (3.6%)	0.75
History of renal problems[FE]	7 (1.6%)	1 (0.3%)	1 (1.7%)	5 (6.0%)	0.003
History of any diabetes[FE]	33 (7.6%)	19 (6.4%)	1 (1.7%)	13 (15.7%)	0.01
History of Hepatitis B Virus	64 (14.7%)	40 (13.6%)	10 (17.2%)	14 (16.9%)	0.63
History of Hepatitis C Virus[FE]	27 (6.2%)	17 (5.8%)	5 (8.6%)	5 (6.0%)	0.69
History of cardiovascular disease	198 (45.4%)	124 (42.0%)	20 (34.5%)	54 (65.1%)	<0.001
History of arthritis of knee or hip	53 (12.2%)	36 (12.2%)	6 (10.3%)	11 (13.3%)	0.80
Sleep Medication					
Sleep medication use for insomnia[FE]	37 (8.5%)	26 (8.8%)	3 (6.9%)	7 (8.4%)	0.97

[FE]Fisher's exact test was conducted due to expected cell counts of less than 5.
[†]p-value for between-cluster differences.
*Note, the following variables were missing data (number of participants missing data overall in parentheses): Obese (5), Systolic Blood Pressure (3), Diastolic Blood Pressure (3), Ever injected drugs (1)

Table S5. Unadjusted and adjusted logistic regression (Insomnia, ISI ≥15) and linear regression (ISI score, continuous) results for (a) analysis that excluded HIV-negative controls and (b) analysis that excluded people living with HIV that experienced viraemia (HIV-RNA >50 copies/mL) at the POPPY baseline visit

Odds ratio (OR (95% confidence interval (CI)) for logistic regression or mean difference (95% CI) for linear regression, unless otherwise noted	**Overall***	**Cluster**			**p†**
		**I**	**II**	**III**	
**(a)** Analysis that excluded HIV-negative controls					
**Logistic Regression Results, *Insomnia (ISI≥15)*, *Estimated OR of Insomnia (95% CI)*****					
n	343	216	39	88	--
Insomnia (ISI ≥15), n (%)	76 (23.2%)	43 (20.7%)	8 (22.2%)	25 (29.8%)	--
Unadjusted	--	**REF**	1.10 (0.44, 2.48)	1.63 (0.91, 2.88)	0.26
Adjusted (HIV status, age, sex, race)	--	**REF**	1.12 (0.45 2.56)	1.62 (0.89, 2.89)	0.28
**Linear Regression Results, ISI Score (continuous), *Estimated Mean Difference in ISI Score (95% CI)*****					
ISI Score, mean (standard deviation (SD))	9.22 (6.35)	8.91 (6.28)	8.92 (6.34)	10.10 (6.50)	--
Unadjusted	--	**REF**	0.00 (-2.25, 2.26)	1.21 (-0.41, 2.82)	0.33
Adjusted (HIV status, age, sex, race)	--	**REF**	0.00 (-2.26, 2.27)	1.28 (-0.36, 2.92)	0.29
**(b)** Analysis that excluded people living with HIV that experienced viraemia (HIV-RNA >50 copies/mL) at the POPPY baseline visit					
**Logistic Regression Results, *Insomnia (ISI≥15)*, *Estimated OR of Insomnia (95% CI)*****					
n	436	295	58	83	--
Insomnia (ISI ≥15), n (%)	73 (17.3%)	48 (16.8%)	10 (17.5%)	15 (19.0%)	--
Unadjusted	--	**REF**	1.05 (0.48, 2.16)	1.16 (0.60, 2.17)	0.90
Adjusted (HIV status, age, sex, race)	--	**REF**	1.44 (0.63, 3.09)	1.08 (0.53, 2.09)	0.67
**Linear Regression Results, ISI Score (continuous), *Estimated Mean Difference in ISI Score (95% CI)*****					
ISI Score, mean (SD)	8.24 (5.96)	8.17 (6.01)	7.91 (5.92)	8.72 (5.87)	--

Table S5. Unadjusted and adjusted logistic regression (Insomnia, ISI ≥15) and linear regression (ISI score, continuous) results for (a) analysis that excluded HIV-negative controls and (b) analysis that excluded people living with HIV that experienced viraemia (HIV-RNA >50 copies/mL) at the POPPY baseline visit

Unadjusted	--	**REF**	-0.26 (-1.96, 1.44)	0.55 (-0.94, 2.04)	0.70
Adjusted (HIV status, age, sex, race)	--	**REF**	0.34 (-1.33, 2.02)	0.53 (-0.98, 2.02)	0.76

[†]p-value from joint (likelihood ratio) test that the coefficients for clusters II and III are both 0.
*Note that 15 individuals were missing data on insomnia/the ISI score in the analysis that excluded HIV-negative controls; and 14 individuals were missing data on insomnia/the ISI score in the analysis that excluded people living with HIV that experienced viraemia (HIV-RNA>50 copies/mL) at the POPPY baseline visit.
**Profile likelihood-based CIs are presented for logistic regression results, and Wald CIs are presented for linear regression results.

Sensitivity Analyses Using Only Individual Log-Transformed Biomarkers

In sensitivity analyses using only individual log-transformed biomarkers (i.e. not using the PCA-AHCA approach), few statistically significant associations were observed with insomnia among the 31 plasma biomarkers; similar results were observed for the linear regression analysis using the ISI score as the outcome. For the logistic regression analysis with insomnia (ISI ≥15) as the outcome, both IL-18 and NFL were statistically significant at the 5% significance level before adjustment, and only NFL remained statistically significant after adjustment (HIV status, age, sex, race). IL-18, a proinflammatory cytokine, had strong positive association with insomnia, while NFL, a polypeptide that is a marker of axonal injury, was estimated to have a strong negative association with insomnia. This negative association with NFL was unexpected, since it was hypothesized NFL would be positively associated with insomnia. For the linear regression analysis with the continuous ISI score as the outcome, only CD163, a macrophage-specific protein that is a marker of innate immune activation, was statistically significant at the 5% significance level before adjustment. CD163 had a moderate positive association with the ISI score (**Table S6**, below). As noted for the primary results, these results should be interpreted with caution due to multiple hypothesis testing. The estimated coefficients for the (log-transformed) biomarkers approximately represent a change in the outcome for a 1% increase in the concentration of a biomarker. So, a change in the odds of insomnia for the logistic regression analysis and mean ISI score for the linear regression analysis for a 1% increase in the concentration of a biomarker.

Table S6. Logistic regression (Insomnia, ISI≥15) and linear regression (ISI score, continuous) results for all 31 individual (log-transformed) biomarkers: unadjusted and adjusted (HIV status, age, sex, race)

		Logistic Regression (Odds ratio (OR) scale)				Linear Regression			
**Inflammatory Pathway**	**Biomarker**	**Crude OR (95% CI)***	**p-value**	**Adjusted OR (95% CI)***	**p-value**	**Crude Estimate (95% CI)***	**p-value**	**Adjusted Estimate (95% CI)***	**p-value**
**Atherosclerosis**	MPO	1.14 (0.84, 1.52)	0.40	1.06 (0.76, 1.45)	0.71	0.35 (-0.37, 1.08)	0.34	0.33 (-0.40, 1.06)	0.37
	lpPla-2	1.38 (0.80, 2.45)	0.26	1.41 (0.81, 2.53)	0.23	0.62 (-0.63, 1.87)	0.33	0.70 (-0.55, 1.95)	0.27
**Coagulation**	D-Dimer	0.96 (0.59, 1.53)	0.86	1.05 (0.62, 1.75)	0.86	-0.29 (-1.41, 0.83)	0.61	-0.05 (-1.21, 1.10)	0.93
	P-Selectin	0.71 (0.35, 1.42)	0.33	0.60 (0.29, 1.24)	0.17	-0.47 (-2.12, 1.18)	0.58	-0.69 (-2.31, 0.93)	0.40
	CD40	1.14 (0.60, 2.11)	0.69	0.92 (0.46, 1.77)	0.80	0.61 (-0.89, 2.11)	0.43	0.11 (-1.38, 1.60)	0.89
	vWF	1.09 (0.77, 1.56)	0.63	0.96 (0.67, 1.40)	0.84	0.63 (-0.18, 1.45)	0.13	0.37 (-0.46, 1.20)	0.38
**Endothelial function**	E-Selectin	0.88 (0.51, 1.54)	0.66	0.78 (0.44, 1.38)	0.39	0.29 (-1.01, 1.60)	0.66	0.10 (-1.18, 1.38)	0.88
	VCAM1	1.30 (0.73, 2.32)	0.37	1.07 (0.59, 1.94)	0.82	0.45 (-0.91, 1.81)	0.51	-0.07 (-1.42, 1.29)	0.92
	ICAM-1	0.91 (0.63, 1.31)	0.61	0.88 (0.60, 1.28)	0.50	0.15 (-0.71, 1.01)	0.73	0.06 (-0.79, 0.91)	0.89
**Innate immune activation**	CD14	1.15 (0.55, 2.43)	0.72	0.99 (0.45, 2.20)	0.98	0.65 (-1.09, 2.40)	0.46	-0.04 (-1.81, 1.73)	0.97
	IL-10	1.17 (0.77, 1.76)	0.45	1.22 (0.79, 1.84)	0.36	-0.05 (-1.05, 0.96)	0.92	0.04 (-0.94, 1.02)	0.94
	MCP-1	1.08 (0.57, 2.01)	0.81	0.92 (0.47, 1.74)	0.79	0.60 (-0.88, 2.08)	0.43	0.23 (-1.23, 1.70)	0.75
	CD163	1.32 (0.90, 1.96)	0.16	1.10 (0.74, 1.66)	0.64	1.21 (0.32, 2.10)	0.01	0.82 (-0.08, 1.72)	0.07
	MIP-1α	1.77 (0.64, 4.74)	0.26	1.50 (0.53, 4.15)	0.43	1.16 (-1.33, 3.65)	0.36	0.81 (-1.65, 3.27)	0.52
**Microbial translocation**	LBP	0.99 (0.71, 1.48)	0.97	1.03 (0.74, 1.51)	0.88	0.27 (-0.57, 1.12)	0.52	0.30 (-0.52, 1.12)	0.47
	IL-18	1.57 (1.01, 2.43)	0.04	1.44 (0.89, 2.30)	0.13	0.83 (-0.24, 1.91)	0.13	0.61 (-0.49, 1.70)	0.28
	IL-12	0.97 (0.71, 1.31)	0.85	1.03 (0.76, 1.41)	0.83	-0.54 (-1.25, 0.17)	0.13	-0.42 (-1.12, 0.28)	0.24
	I-FABP	0.98 (0.85, 1.13)	0.73	0.92 (0.78, 1.08)	0.30	-0.07 (-0.41, 0.28)	0.69	-0.17 (-0.51, 0.17)	0.32
**Systemic inflammation**	CRP	1.08 (0.89, 1.32)	0.42	1.04 (0.85, 1.28)	0.69	0.24 (-0.21, 0.69)	0.30	0.18 (-0.27, 0.63)	0.43
	IFN-γ	1.07 (0.78, 1.46)	0.67	1.16 (0.83, 1.61)	0.37	-0.30 (-1.06, 0.45)	0.43	-0.16 (-0.91, 0.58)	0.67
	TNF-α	1.00 (0.58, 1.67)	0.99	1.14 (0.65, 1.96)	0.64	-0.24 (-1.49, 1.00)	0.70	-0.01 (-1.23, 1.22)	0.99
	IL-6	1.21 (0.82, 1.79)	0.33	1.23 (0.83, 1.83)	0.30	0.24 (-0.69, 1.18)	0.61	0.32 (-0.61, 1.25)	0.50
	IL-2	0.96 (0.76, 1.21)	0.72	1.04 (0.81, 1.33)	0.76	-0.37 (-0.92, 0.17)	0.18	-0.30 (-0.85, 0.24)	0.27
	IL-1β	0.92 (0.63, 1.31)	0.64	0.98 (0.66, 1.42)	0.91	-0.53 (-1.38, 0.32)	0.22	-0.43 (-1.26, 0.40)	0.31
	TNF RII	0.93 (0.50, 1.74)	0.82	0.81 (0.42, 1.55)	0.52	0.77 (-0.69, 2.24)	0.30	0.62 (-0.85, 2.10)	0.41
	TNF RI	0.83 (0.37, 1.82)	0.64	0.76 (0.34, 1.73)	0.52	1.19 (-0.68, 3.05)	0.21	1.26 (-0.62, 3.14)	0.19
**Immune regulation**	PDL-1	1.37 (0.63, 2.91)	0.42	1.05 (0.46, 2.29)	0.91	0.26 (-1.57, 2.09)	0.78	-0.39 (-2.26, 1.48)	0.68
	IL-4	0.92 (0.61, 1.36)	0.68	1.01 (0.65, 1.51)	0.98	-0.45 (-1.39, 0.48)	0.34	-0.39 (-1.32, 0.54)	0.41
	IL-1RA	1.34 (0.88, 2.02)	0.16	1.35 (0.88, 2.07)	0.17	0.71 (-0.29, 1.71)	0.16	0.79 (-0.19, 1.77)	0.11
**Axonal injury**	NFL	0.54 (0.34, 0.85)	0.01	0.48 (0.27, 0.83)	0.01	-0.73 (-1.76, 0.30)	0.16	-0.65 (-1.80, 0.50)	0.26
	S100B	0.86 (0.43, 1.62)	0.64	0.80 (0.38, 1.59)	0.54	0.29 (-1.22, 1.80)	0.71	0.56 (-0.98, 2.10)	0.47
